# Single-Cell Sequencing Reveals γδT Cell Heterogeneity Under Distinct Microsatellite Statuses as a Potential Biomarker for Immunotherapy and Prognosis in Colorectal Cancer

**DOI:** 10.3390/genes17040387

**Published:** 2026-03-29

**Authors:** Xingnuo Zhu, Qi Cao, Yan Ge, Xinyan Zhao, Zhongsheng Sun

**Affiliations:** 1 Institute of Genomic Medicine, Wenzhou Medical University, Wenzhou 325000, China; zhuxn@wmu.edu.cn (X.Z.); 13628667715@163.com (Q.C.); gy1978185574@163.com (Y.G.); 2 Hangzhou Institute of Medicine (HIM), Chinese Academy of Sciences, Hangzhou 310022, China; 3 School of Life Sciences, Tianjin University, Tianjin 300072, China; zhaoxinyan_zxy@163.com

**Keywords:** colorectal cancer, γδT cell, microsatellite instability, single-cell RNA sequencing

## Abstract

**Background**: Colorectal cancer (CRC) continues to represent one of the most common and lethal malignant tumors globally. Notably, only patients diagnosed with microsatellite instability-high (MSI-H) colorectal cancer derive substantial clinical benefits from immune checkpoint inhibitor therapy. As critical immune cells that infiltrate tumors, γδT cells are tightly linked to the therapeutic response in colorectal cancer patients with microsatellite instability (MSI) colorectal cancer. However, the heterogeneous characteristics of γδT cells in colorectal cancer with different microsatellite statuses and their specific roles in regulating immunotherapy responses remain unclear. **Methods**: We performed dimensionality reduction and clustering analysis on γδT cells from a single-cell RNA sequencing dataset to explore diversity and functional characteristics of distinct γδT cell subsets. Meanwhile, bulk transcriptome data were applied to further investigate the immune infiltration, clinical characteristics, and immune checkpoint molecule expression in CRC patients stratified by distinct γδT cell subpopulations. **Results**: We identified five γδT cell subsets, among which the C4_CXCL13 γδT cell subsets was enriched in MSI CRC and exhibited an exhausted-like T cell phenotype while retaining robust cytotoxic function. A signature score based on these 17 marker genes was associated with survival, immune infiltration, and therapeutic response, thus representing a potentially valuable independent prognostic factor. **Conclusions**: The C4_CXCL13 γδT cell subset represents a characteristic subset in MSI CRC and is closely associated with clinical prognosis and benefit from immunotherapy. It represents a potential clinical marker for classifying patients and estimating the response to immunotherapy, offering a novel target for personalized immunotherapy in CRC.

## 1. Introduction

Colorectal cancer (CRC) ranks third in incidence and second in mortality among all malignant tumors globally [1]. CRC can be classified into two main molecular subtypes: dMMR/MSI-H (∼15% of patients) and MSS/MSI-L, which accounts for the vast majority of cases (85%) [2]. Accumulating clinical trials for colorectal cancer have demonstrated remarkable success of immune checkpoint blockade (ICB), with particularly pronounced benefits observed in MSI or dMMR patients [3,4]. Although MSI-high status is widely used to predict ICB response [5], not all MSI-high patients achieve durable clinical benefits, and pathological responses have also been observed in some MSS colon tumors [6]. Therefore, a comprehensive dissection of cellular and molecular dynamics in CRC with distinct microsatellite statuses will contribute to the establishment of innovative strategies, ultimately benefiting a broader range of CRC patients.

Previously, immunotherapies for CRC primarily targeted αβ T cells, which exert anti-tumor cytotoxicity through a histocompatibility complex (MHC)-dependent pathway [7]. However, cancer cells frequently downregulate or lose MHC expression, thereby escaping from αβT cell-mediated immune surveillance and killing [8]. In contrast, γδT cells display MHC-unrestricted cytotoxicity against various tumor cells in vitro, highlighting their promising potential for cancer immunotherapy [9].

In our study, we jointly analyze single-cell and bulk RNA sequencing data from CRC patients to classify γδT cell subsets, elucidate the diversity and functional characteristics of γδT cells in CRC patients with different microsatellite statuses, identify γδT cell subsets with significant clinical relevance, and explore their clinical features and association with immunotherapy response. Our findings aim to identify novel γδT cell biomarkers for predicting immunotherapy response in colorectal cancer and lay a foundation for the development of γδT cell-targeted interventions.

## 2. Materials and Methods

### 2.1. Single-Cell RNA-Seq Data Collection and Preliminary Processing

The colorectal cancer scRNA-seq dataset GSE178341 [10] was retrieved from the GEO database: https://www.ncbi.nlm.nih.gov/ (accessed on 20 January 2026), comprising 29 MSI CRC tumor samples, 35 MSS CRC tumor samples and 36 adjacent tissue samples. Further analyses of the scRNA-seq data were conducted using Scanpy [11] (v1.10.1) within the Python environment (v3.11). Initially, genes expressed in fewer than three cells were excluded, and cells with fewer than 200 detected genes were removed. The proportion of mitochondrial reads was quantified by identifying genes whose names began with “MT-” via the ‘var_names.str.startswith (‘MT-’)’ function. Gene expression levels within each cell were normalized to the total read count and subsequently log-transformed. Following these preprocessing steps, batch-aware centering and scaling were applied across all samples using default parameters. The top 2000 highly variable genes (HVGs) were then identified using the “sc.pp.highly_variable_genes” function and utilized for subsequent downstream analyses.

### 2.2. Dimensionality Reduction and Cluster Identification

Cellular partitions were identified using multiple rounds of iterative leiden clustering via the “scanpy.tl.leiden” function. T-distributed stochastic neighbor embedding (t-SNE) was performed to visualize cell clusters and validate batch correction effects. Cell clusters were annotated according to the expression of canonical marker genes. The processed AnnData object in Python (v3.11) was exported in a H5AD format and converted to a Seurat object in R (v4.2.3) using the “h5ad2seurat” function from the schard (v0.0.1) package. Differentially expressed genes (DEGs) for each cluster were identified using the “sc.tl.rank_genes_groups” function with default parameters and the Wilcoxon rank-sum test.

γδT cells were identified according to the expression of signature genes, including *CD3D*, *CD3E*, *TRDC*, *TRGC1*, *TRGC2* [12,13]. The “FindAllMarkers” function implemented in the Seurat [14] package was (v4.2.3) applied to identify cluster-specific marker genes.

### 2.3. Functional Enrichment Analysis

Functional enrichment investigations, including Gene Ontology (GO) terms and Kyoto Encyclopedia of Genes and Genomes (KEGG) pathways, were implemented using the clusterProfiler package (v4.7.1.3) [15]. Statistical significance was determined for enriched terms at a *p*-value < 0.05 by means of “enrichGO” and “enrichKEGG” functions. The inherent “GSEA” function within clusterProfiler was applied to carry out gene set enrichment analysis.

### 2.4. Signature Score Calculation

Differentially expressed genes of γδT cell subsets were identified using the “FindAllMarkers” function with thresholds: avg_log2FC > 0.58, *p*_val_adj < 0.05, and pct.1 > 0.3. Univariate and multivariate Cox regression analyses were then performed, and the “AddModuleScore”function (default parameters) was applied to calculate the characteristic scores for verification. Ultimately, a gene set consisting of 17 genes was identified as a prognostic marker. The boxplot of the score expression was generated using the “geom_boxplot” function in the ggplot2 package.

### 2.5. Pseudotime Analysis

CytoTRACE [16] package (v1.0.0) was employed to quantify the differentiation capacity of individual cell states and determine the sequential order of cell trajectories. Pseudotime dynamics of γδT subpopulations were interrogated via the Monocle [17] package (v2.30.0). DEGs across pseudotime during cellular transitions were detected with the “differentialGeneTest” function (q-value < 0.1). The “DDRTree” function was adopted for dimensionality reduction, and trajectory plots were generated using the “plot_cell_trajectory” function.

### 2.6. Copy Number Variation Analysis

Malignant cells within the epithelial cell population were identified by estimating copy number variations (CNVs) via the infercnvpy algorithm (https://github.com/icbi-lab/infercnvpy, accessed on 20 January 2026). Fibroblasts and endothelial cells were adopted as normal control cells during algorithm execution, with all parameters set to default. Single-cell CNV scores were subsequently computed using the “infercnvpy.tl.cnv_score” function.

### 2.7. Cell Communication Analysis

Analysis of cell–cell crosstalk networks was performed using the CellChat [18] package (v1.6.1). The “createCellChat” function was utilized to build a CellChat object, and the “identifyOverExpressedInteractions” function was adopted to screen overexpressed ligands and receptors for PPI network establishment. The communication probabilities and intercellular communication networks were further predicted using the “computeCommunProb” function. Cellular interactions and corresponding signaling pathways were visualized with the “netVisual_bubble” function.

### 2.8. RNA-Seq Data Collection and Preprocessing

Bulk transcriptomic profiles and relevant clinicopathological information of CRC were collected from the TCGA database (http://www.cancer.gov/tcga, accessed on 20 January 2026). Samples with confirmed MSS or MSI status were enrolled, yielding a count matrix covering 425 specimens. The dataset included a count matrix comprising 425 samples. Simple nucleotide variation data for colon adenocarcinoma were acquired from the cBioPortal (https://www.cbioportal.org/datasets, accessed on 20 January 2026). The R package MAFtools [19] (v2.14.0) was applied to calculate tumor mutational burden (TMB) values, denoted as the mutation count per megabase. For external validation, the GSE39582 [20] dataset including 558 colon cancer tissues and IMvigor210 cohort data were obtained from http://research-pub.gene.com/IMvigor210CoreBiologies (accessed on 20 January 2026), with 298 patients stratified by immunotherapy response enrolled. Immunophenoscores (IPS) were obtained from the TCIA database (https://tcia.at/, accessed on 20 January 2026) to evaluate the relationship between risk scores and immunotherapy outcomes.

### 2.9. Gene Set Variation Analysis

Signature scores for 17 C4_CXCL13 γδT cell-associated genes were calculated using the R package GSVA [21] (v1.50.0) to quantify the abundance of C4_CXCL13 γδT cells per sample. Based on the C4_CXCL13 γδT cell scores, all samples were assigned to two distinct groups.

### 2.10. Analysis of Immune Cell Infiltration

Stromal, immune, and ESTIMATE scores were computed with the R package ESTIMATE to characterize the TME and evaluate tumor purity in COAD patients [22]. Based on a published panel of 28 immune cell subsets, the immune infiltration profiles of each sample were further analyzed using the ssGSEA approach. The CIBERSORT [23] package (v0.1.0) was employed to estimate the fractions of 22 tumor-infiltrating immune cells via deconvolution based on the LM22 immune feature matrix.

### 2.11. Statistical Analysis

The survminer package (v0.4.9) with its “surv_cutpoint” function was utilized to calculate the optimal cutoff of the signature score [24]. Survival curves were plotted using the Kaplan–Meier approach, and intergroup differences were assessed via the log-rank test. All statistical analyses, including the Wilcoxon rank-sum test, Chi-square test, and log-rank test, were carried out in R (v4.1.0) and Python (v3.11). Statistical significance was defined as: *p* < 0.05 (*), *p* < 0.01 (**), *p* < 0.001 (***), and *p* < 0.0001 (****).

## 3. Results

### 3.1. Single-Cell Landscape of MSI and MSS CRC

**Figure 1:**
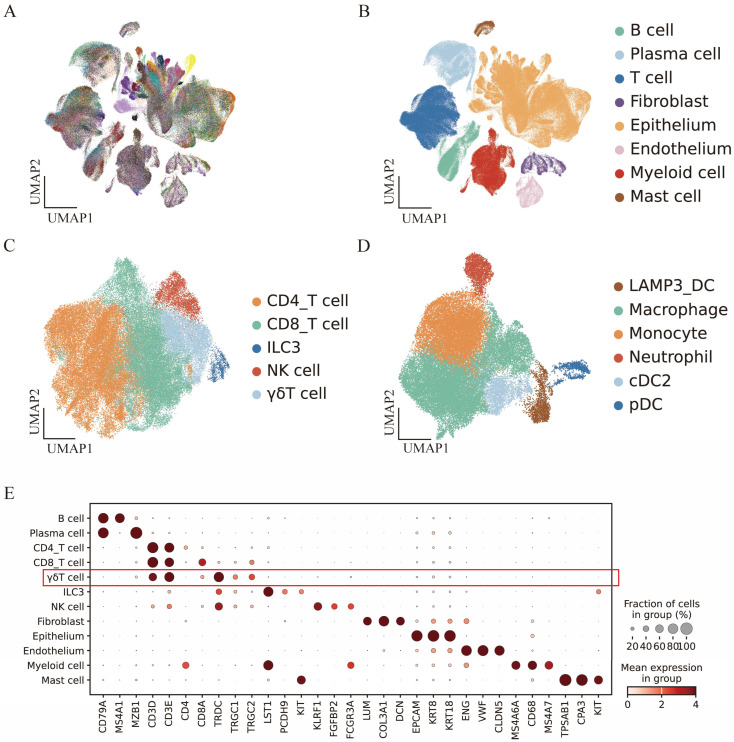
Landscape of cellular infiltrates in MSI and MSS colorectal cancer. (**A**) UMAP clusters are labeled based on patients. (**B**) UMAP clusters are annotated according to main cell lineages. (**C**,**D**) UMAP plots of the T/NK and Myeloid cell compartments colored and labeled by subcluster annotations. (**E**) Dotplot showing expression patterns of marker genes across all cell clusters. Dot size reflects the percentage of cells expressing the gene, and color represents the average expression level. The red box marks the γδT cell cluster, verifying its identity via characteristic gene expression.

Single-cell transcriptomic profiling of 100 CRC specimens from the GSE178341 dataset was performed to delineate infiltrating cell populations in MSI and MSS CRC. Microsatellite statuses for all patients in the cohort are provided in Supplementary Materials, Table S1. Following stringent quality control and removal of low-quality cells, 360,282 cells were preserved for scRNA-seq analysis (Figure 1A and  Figure S1). Using canonical cell-type markers, these cells were annotated into 8 major cell lineages: T/NK cells (*CD3D*, *CD3E* and *FCGR3A*), B cells (*CD79A*, *MS4A1*), plasma cells (*CD79A*, *MZB1*), myeloid cells (*MS4A6A*, *LYZ* and *CD68*), fibroblasts (*DCN*, *LUM*), endothelial cells (*ENG*, *PECAM1* and *CLDN5*), epithelial cells (*EPCAM*, *KRT8* and *KRT18*), and mast cells (*TPSAB1*, *CPA3* and *KIT*) (Figure 1B). We then evaluated the status and composition of cell clusters in the adjacent tissues, MSI CRC tissues, and MSS CRC tissues. Given the prevalence of immune cells in CRC tumors and their anti-tumor capacities, we further subdivided the T/NK cells and myeloid cells into subsets (Figure 1C,D). For T/NK cell subsets, γδ T cells were identified and isolated in light of the expression of the cell type-specific marker genes *TRDC*, *TRGC1*, and *TRGC2*, rather than marker genes for CD4^+^ or CD8^+^ T cells. Established cell type-specific markers were employed to manually annotate every cell cluster (Figure 1E).

### 3.2. Identification of γδT Cluster Associated with Tumors

**Figure 2:**
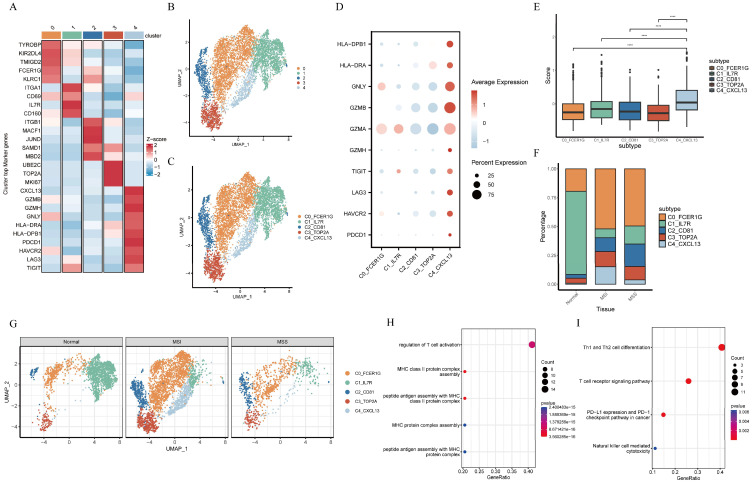
Heterogeneity of γδT cells in MSI and MSS CRC revealed by single-cell RNA-seq. (**A**) Heatmap of signature genes across different cell clusters. (**B**) UMAP embedding of the distribution of γδT cells in MSI and MSS CRC. (**C**) UMAP visualization of γδT cell clusters after annotation. (**D**) Dotplot showing the expression of cytotoxic and immune checkpoint genes. (**E**) Boxplots showing the immune checkpoint score of γδT cell subsets (**** *p* < 0.0001). (**F**) Distribution of γδT cell subset fractions across tissues. (**G**) UMAP embedding of γδT cell clusters from adjacent tissue, MSS CRC, and MSI CRC. (**H**) Gene Ontology and pathway enrichment analysis of DEGs upregulated in C4_CXCL13 γδT cells. (**I**) KEGG enrichment analysis of DEGs upregulated in C4_CXCL13 γδT cells.

We extracted all cells annotated as γδT cells from the integrated single-cell dataset. γδT cells clustered into five clusters (Figure 2B). Cluster identity was determined by signature genes of T cell subsets and functional states (Figure 2A,C and Supplementary Materials Table S2). The highest percentage of effector C0_FCER1G γδT cell subset, along with elevated expression of *TYROBP*, natural killer cell surface receptor-related genes (*KLRC1*, *KIR2DL4*), and innate immune-related molecules (*FCER1G* [25], *TMIGD2*) were found in tumor tissue from CRC patients, indicating enhanced expression of genes associated with the intraepithelial lymphocyte (IEL) [26]. In the adjacent tissue samples, the C1_IL7R γδT cell subset showed higher expression levels of tissue-resident genes (*ITGAE* and *CD69*) [13], and given its abundant *IL7R* expression, this subset was identified as a potential memory γδT cell subset. The C2_CD81 γδT cell subset expressed *SAMD1*, *MBD2*, and *JUND*, whose encoded proteins confer resistance to oxidative stress, while also exhibiting high expression of multiple adhesion molecules including *ITGB1* and *MACF1*. The C3_TOP2A γδT cell subset was characterized by a high expression of mitotic-related genes (*TOP2A*, *UBE2C*, *MKI67*). The C4_CXCL13 γδT cell subset displayed markedly elevated expression of cytotoxic genes (*GZMA*, *GZMB*, *GZMH*, *PRF1*) and antigen presentation-related genes (*HLA-DRA*, *HLA-DPB1*).

Relative to other γδT cell subsets, the C4_CXCL13 γδT cells specifically expressed *PDCD1* and *CXCL13*, along with distinct upregulation of multiple immune checkpoint molecules, including *LAG3*, *TIGIT*, and *HAVCR2*. Moreover, the C4_CXCL13 γδT cell subset exhibited the highest immune checkpoint score among all subsets (Figure 2D,E). These observations indicate that the C4_CXCL13 γδT cell subset exhibits phenotypic characteristics similar to exhausted T cells yet still retains cytotoxic functions. We found that the proportion of the C4_CXCL13 γδT cell subset was significantly higher in MSI CRC compared with MSS CRC or adjacent tissue. Furthermore, the C4_CXCL13 γδT subset was distinct from other γδT cell subsets, representing a unique population specifically identified in MSI CRC (Figure 2F,G). To comprehensively characterize the biological roles of C4_CXCL13 γδT cells, enrichment analysis was performed. Gene Ontology (GO) enrichment analysis indicates that the C4_CXCL13 γδT cell subset was significantly enriched in pathways associated with antigen presentation, αβ T cell activation, and leukocyte-mediated cytotoxicity (Figure 2H). Additionally, KEGG pathway analysis further demonstrated prominent enrichment in tumor PD-L1 expression and the PD-1 checkpoint pathway (Figure 2I).

### 3.3. Trajectory Analysis of γδT Cell Subsets in Colorectal Cancer

**Figure 3:**
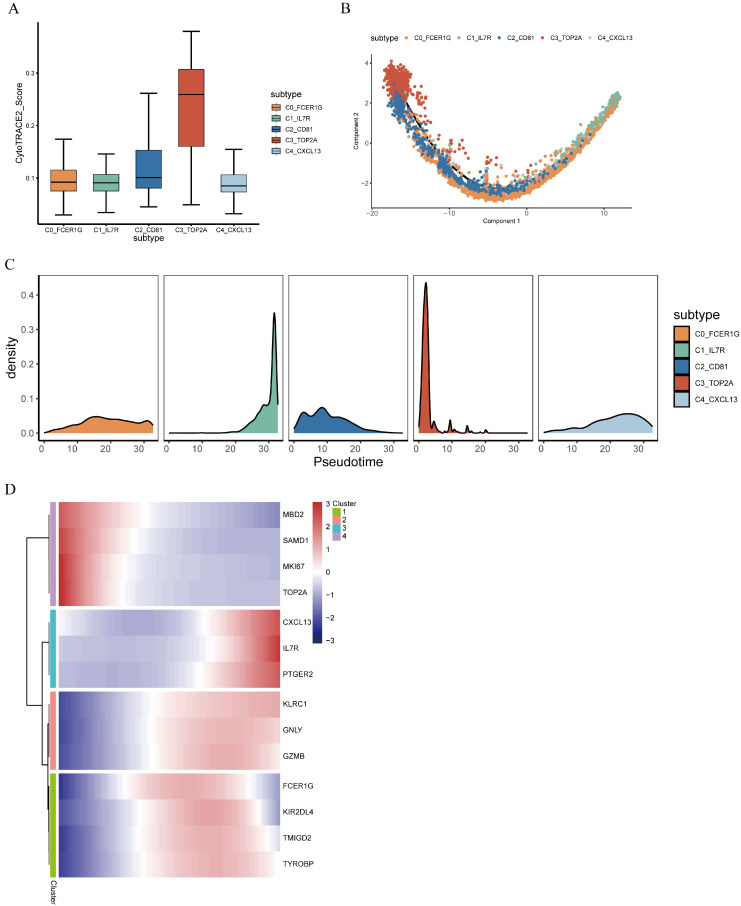
γδT cell subsets pseudotime analysis. (**A**) Boxplots showing the differentiation potential scores of γδT cells. (**B**) Color visualization of γδT cell subsets according to pseudotime. (**C**) Cell density variation in γδT cells during pseudotime. (**D**) Heatmap showing dynamic shifts in gene expression along the trajectory.

CytoTRACE analysis was used to evaluate the differentiation capacity of distinct γδT cell subsets, with results showing that the C3_TOP2A γδT cell subset displayed the highest CytoTRACE score, indicating the lowest degree of differentiation (Figure 3A). We analyzed the differentiation trajectories of γδT cell subsets using pseduotime analysis and observed the developmental trajectories from C3_TOP2A γδT cells at the initial state or C2_CD81 γδT cells at the intermediate state to C4_CXCL13 γδT cells and C1_IL7R γδT cells at the terminal state (Figure 3B,C). Based on pseduotime calculations, it was speculated that the high proliferating C3_TOP2A γδT cell subset mainly differentiated into the effector C4_CXCL13 γδT subset and the memory C1_IL7R γδT cell subset, whereas the C0_FCER1G γδT cells were distributed throughout the trajectory and the C2_CD81 γδT cell subset was mainly located in the intermediate transition state. We performed pseudotime analysis to investigate the dynamic expression profiles of pivotal genes during γδT cell differentiation. Differentially expressed genes were clustered into four groups with similar temporal expression profiles (Figure 3D). Cluster 4 was mainly located in the early pseudotime stage, characterized by marker genes derived from the C3_TOP2A and C2_CD81 γδT cell subsets. Clusters 2 and 3 were predominantly distributed in the terminal stage, harboring marker genes of the C1_IL7R and C4_CXCL13 γδT cell subsets.

### 3.4. Cell–Cell Interaction Analysis of C4_CXCL13 γδT Cells

**Figure 4:**
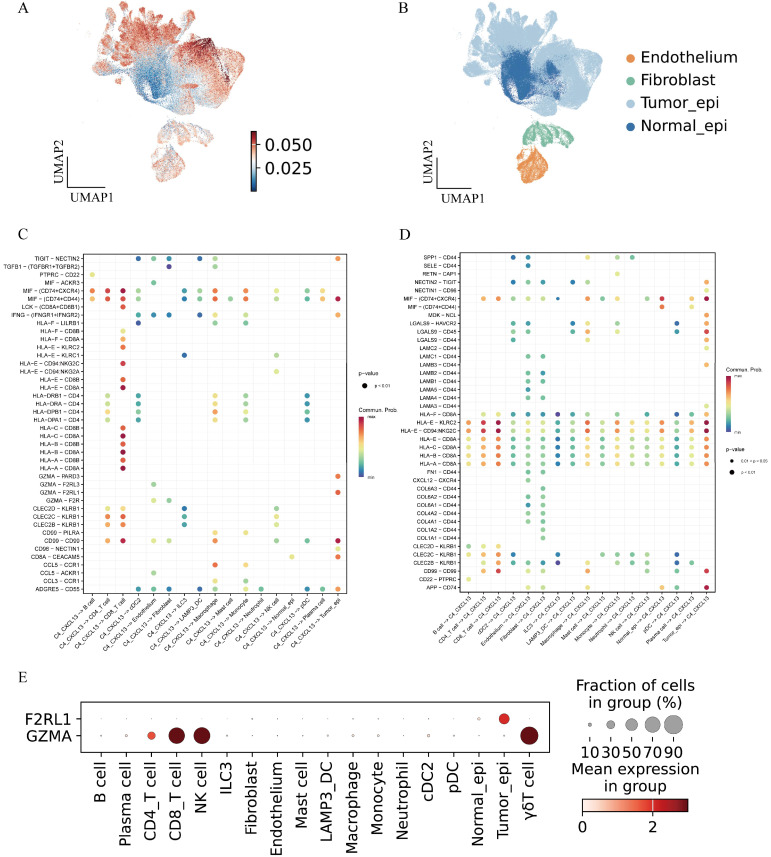
Cell–cell interaction analysis of C4_CXCL13 γδT cells. (**A**) UMAP plot showing the epithelial cells are divided into tumor cells and normal epithelial cells. (**B**) UMAP plot of epithelial cells, colored by inferred CNV score. (**C**) Dotplot showing cell–cell interaction from C4_CXCL13 γδT cells to other cells. (**D**) Dotplot showing cell–cell interaction from other cells to C4_CXCL13 γδT cells. (**E**) Dotplot summarizing the expression status of GZMA and F2RL1 across cell populations.

Next, we conducted cell communication analyses to characterize the ligand–receptor interactions between C4_CXCL13 γδT cells and other cells. To identify the malignant epithelial cells, we included a fibroblasts cluster and an endothelial cells cluster in the analyses as reference cells (Figure 4A). Epithelial cells were further categorized into malignant and non-malignant cells based on distinct CNV signatures. (Figure 4B). We next used CellChat to analyze cell–cell communication between C4_CXCL13 γδT cells and other cell types in MSI CRC and analyzed the cellular signal regulatory events in which C4_CXCL13 γδT cells participate as signal senders and signal receivers, respectively (Figure 4C,D). The results show that CD99-CD99 and MIF-(CD74+CXCR4) are the main intercellular interaction pathways mediated by C4_CXCL13 γδT cells. Accumulating evidence confirms that CD99 is ubiquitously expressed across multiple cell populations, including T cells, myeloid cells, fibroblasts, and epithelial cells. Its core functions involve regulating cell adhesion, transendothelial migration, and multiple immune signaling pathways [27]. In addition, C4_CXCL13 γδT cells can interact specifically with CD4^+^ T cells and myeloid cells through classical class II human leukocyte antigen (HLA) molecules such as HLA-DR and HLA-DP, participating in the regulation of the immune microenvironment. Meanwhile, the chemokines secreted by C4_CXCL13 γδT cells can regulate the function of macrophages through the CCL3-CCR1 and CCL5-CCR1 signaling pathways. Further studies found that C4_CXCL13 γδT cells can interact with tumor cells through receptor–ligand pairs involved in protease-activated receptors (PARs) and midkine (MK) signaling pathways, specifically including MDK-NCL, IFNG-(IFNGR1+IFNGR2), GZMA-PARD3 and GZMA-F2RL1 (*p* < 0.01). Verification of the expression profiles of the above receptor–ligand pairs in different cell types revealed that the F2RL1 molecule preferentially expressed in tumor cells rather than other cell populations (Figure 4E). In summary, the GZMA-F2RL1 signaling axis may represent an important communication pathway between C4_CXCL13 γδT cells and tumor cells, with potential regulatory implications in colorectal cancer development.

### 3.5. Identification of Genes Related to C4_CXCL13 γδT Cells in MSI CRC and GSVA Score Calculation

**Figure 5:**
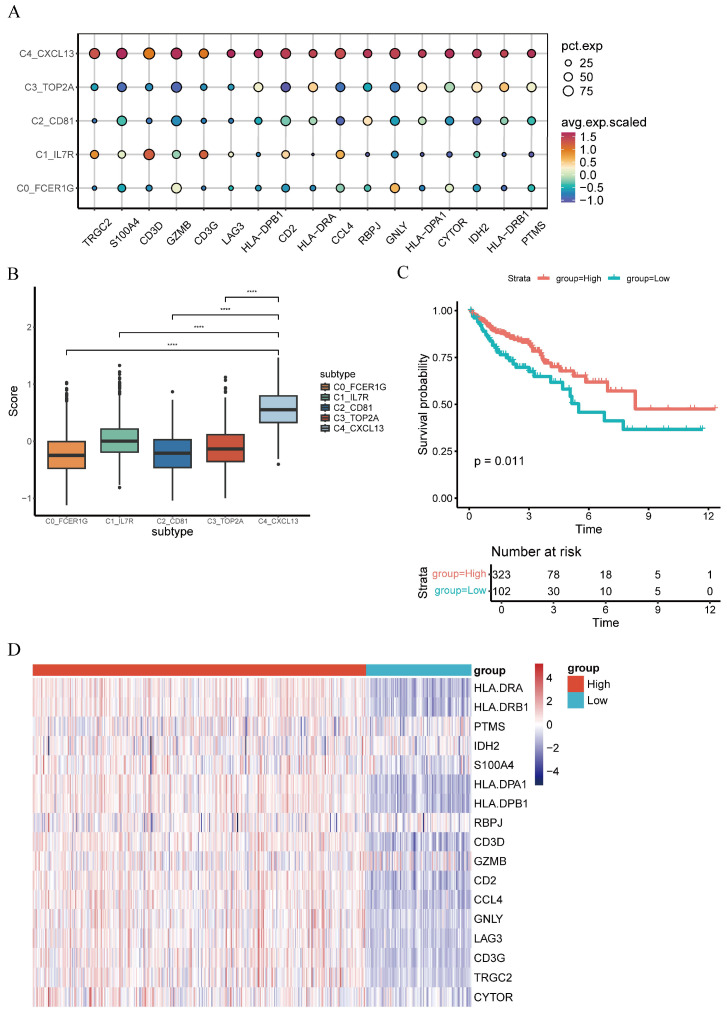
Establishment of the C4_CXCL13 γδT cell score and clustering analysis of C4_CXCL13 γδT cells. (**A**) Dotplot showing the expression of C4_CXCL13 γδT cell subset marker gene. (**B**) Boxplot showing the distribution of C4_CXCL13 γδT cell signature score (**** *p* < 0.0001). (**C**) Prognostic value of C4_CXCL13 γδT cell score in CRC patients. (**D**) Heatmap for patient stratification using the C4_CXCL13 γδT cell score.

To better characterize genes of C4_CXCL13 γδT cells, we performed marker gene screening using the FindAllMarkers function. The screening thresholds were set as follows: *p*_val_adj < 0.05, avg_log2FC > 0.58, and the percentage of cells expressing the gene in the C4_CXCL13 γδT cell subset (pct.1) > 0.3. Ultimately, 17 eligible characteristic genes (*TRGC2*, *S100A4*, *CD3D*, *GZMB*, *CD3G*, *LAG3*, *HLA-DPB1*, *CD2*, *HLA-DRA*, *CCL4*, *RBPJ*, *GNLY*, *HLA-DPA1*, *CYTOR*, *IDH2*, *HLA-DRB1*, *PTMS*) were identified, which were highly expressed in the C4_CXCL13 γδT cell subset (Figure 5A). The results confirm that the gene characteristics of C4_CXCL13 γδT cells have significant specificity among different cell subsets (Figure 5B). We next performed GSVA analysis to generate C4_CXCL13 γδT cell signature score across all samples in TCGA-COAD, and samples were assigned to either a high-score group or a low-score group with 323 cases assigned to the high-score group and 102 to the low-score group (Figure 5D). As shown by Kaplan–Meier analysis, increased C4_CXCL13 γδT cell signature scores were significantly linked to improved survival in the TCGA-COAD cohort (Figure 5C). Subsequently, we conducted univariate and multivariate Cox analyses based on the C4_CXCL13 γδT cell score and other clinical factors and found that the C4_CXCL13 γδT cell score was a significant independent prognostic factor (*p* < 0.05, Table 1).

### 3.6. Relationship of C4_CXCL13 γδT Cell Signature with Tumor Immune Characteristics

**Figure 6:**
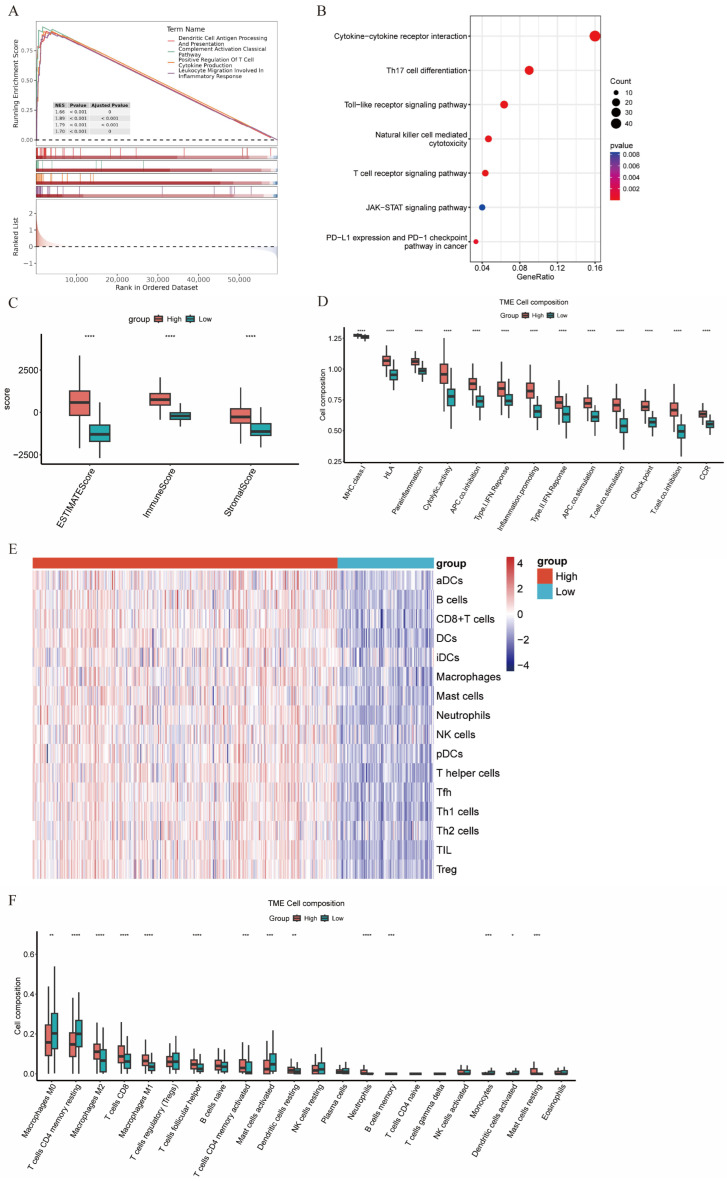
Immune cell infiltration profiles in H-CXCL13 γδT and L-CXCL13 γδT groups from TCGA-COAD. (**A**) GSEA revealing significant enrichment of multiple immune-related terms in the H-CXCL13 γδT group. (**B**) Dotplot showing the enriched KEGG pathways in the H-CXCL13 γδT groups. (**C**) ESTIMATE analysis showing that H-CXCL13 γδT has higher ESTIMATE, immune and stromal scores than L-CXCL13 γδT groups. (**D**) Differences in immune activity between H-CXCL13 γδT and L-CXCL13 γδT groups. (**E**) Heatmap displaying elevated immune cell infiltration in the H-CXCL13 γδT group compared with the L-CXCL13 γδT group. (**F**) The relative abundance of immune cells between two groups (ns = not significant, * *p* < 0.05, ** *p* < 0.01, *** *p* < 0.001, **** *p* < 0.0001).

We screened for DEGs between the High-CXCL13 γδT (H-CXCL13 γδT) group and Low-CXCL13 γδT (L-CXCL13 γδT) group in TCGA-COAD; 457 genes exhibited elevated expression in the H-CXCL13 γδT group. GSEA was performed to explore functional disparities between the two groups, and genes in the H-CXCL13 γδT group were primarily involved in immune-associated biological processes, including leukocyte migration, humoral immune response, and the activation and regulation of adaptive immune responses (Figure 6A). Subsequently, we performed KEGG enrichment analysis on genes with significantly elevated expression in the high-score group and visualized the enrichment results (Figure 6B). The results demonstrated that the upregulated genes were mainly enriched in multiple anti-tumor immune-related pathways, including the cytokine signaling pathway, T cell receptor signaling pathway, PD-L1 immune checkpoint pathway, and Th17 cell differentiation pathway. Collectively, these results indicate that DEGs between the two score groups are mainly localized to immune-related biological activities, suggesting that the H-CXCL13 γδT group may be closely associated with the immune activation status in colorectal cancer.

Immune infiltration analysis results showed that scores associated with the C-C chemokine receptor (CCR) signaling pathway, immune checkpoint scores, MHC class molecule-related scores, and cytotoxic activity response scores were all significantly elevated in the high-score group in comparison with the low-score group (Figure 6D). Furthermore, ssGSEA showed that the level of immune cell infiltration in the H-CXCL13 γδT group was higher than that in the L-CXCL13 γδT group (Figure 6E). These results highlight that an enhanced anti-tumor immune response exists in the tumor microenvironment of patients with high C4_CXCL13 γδT signature scores. To further clarify the differences in immune cell composition within the tumor microenvironment between the two groups, we analyzed the relative proportions of various immune cells in samples from both groups using the CIBERSORT algorithm. The results suggest a significantly higher relative abundance of M2 macrophages, CD8^+^ T cells, follicular helper T cells, M1 macrophages, resting mast cells, and neutrophils in the H-CXCL13 γδT group versus the L-CXCL13 γδT group. In contrast, the relative abundances of M0 macrophages, resting memory CD4^+^ T cells, and activated mast cells were significantly decreased (Figure 6F). In addition, the H-CXCL13 γδT group displayed markedly higher immune and ESTIMATE scores, supporting that C4_CXCL13 γδT cells exert a regulatory effect on immune cell infiltration within the TME (Figure 6C).

### 3.7. Evaluation of Immunotherapy Response Prediction

**Figure 7:**
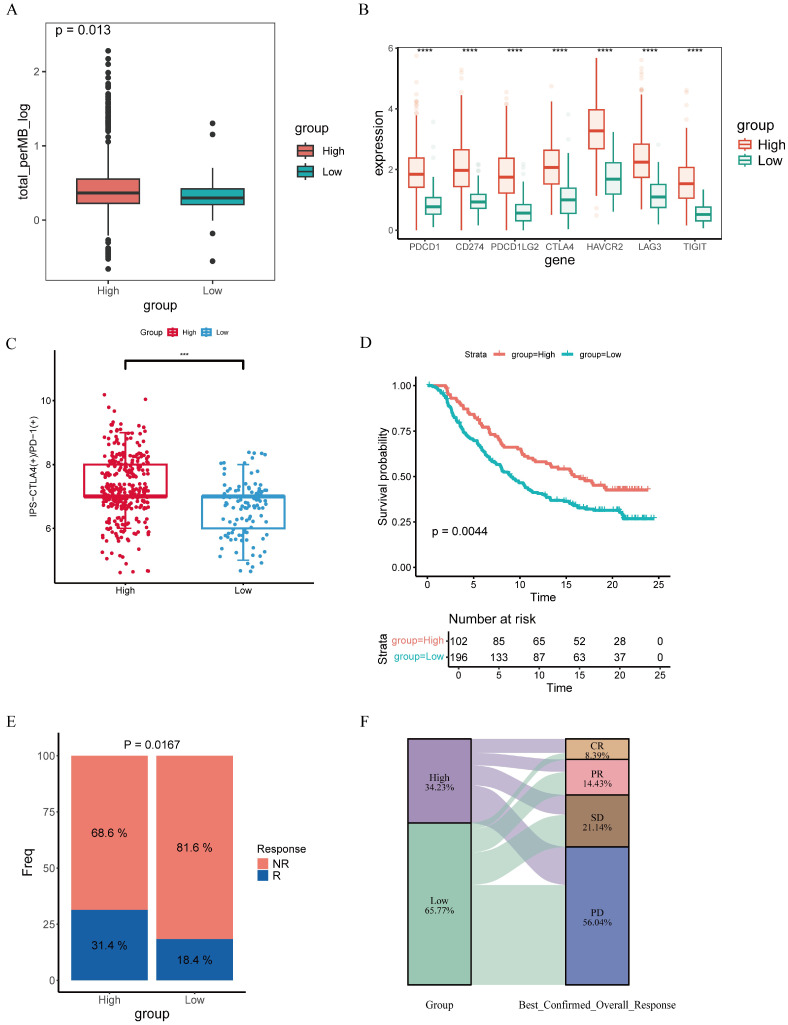
Characterization of immunotherapy responsiveness in TCGA-COAD patients. (**A**) Differential analysis of TMB profiles in the two groups. (**B**) Immune checkpoint expression across the two groups (**** *p* < 0.0001). (**C**) Boxplot showing the difference in immune phenotype score (IPS) in two groups (*** *p* < 0.001). (**D**) Kaplan–Meier curves of high and low C4_CXCL13 γδT score groups in the IMvigor210 cohort. (**E**) The clinical response rates of two groups in response to immunotherapy. (**F**) Sankey diagram illustrating the correspondence of C4_CXCL13 γδT scores with immunotherapy response.

We next evaluated immunotherapy-related characteristics by comparing mutation profiles across groups (Supplementary Materials, Figure S2). The H-CXCL13 γδT group presented higher TMB, which might enhance neoantigen generation and immune activation (Figure 7A). Consistently, major immune checkpoint molecules (*PD1*, *PDL1*, *PDL2*, *CTLA4*, *LAG3*, *HAVCR2*, *TIGIT*) exhibited significantly elevated expression levels in the high-score group (Figure 7B). We next evaluated whether the C4_CXCL13 γδT cell signature could predict immunotherapy responsiveness in patients stratified by microsatellite status (MSI versus MSS). For each group, we separately estimated the abundance of infiltrating immune cells and the transcript levels of immune checkpoint molecules across individual samples. Our analysis revealed that within both MSI and MSS groups, patients in the H-CXCL13 γδT group had higher immune infiltration and immune checkpoint expression, as well as higher activated CD8^+^ T cell infiltration and higher levels of type I interferons—these features were associated with better immune treatment responses and prognosis (Supplementary Materials, Figure S3). Subsequently, we then looked for any correlation between C4_CXCL13 γδT cell signature scores and immunotherapeutic response. We performed Immunophenotype Score (IPS) analysis for anti-CTLA-4 and anti-PD-1 in samples from both the high- and low-score groups to predict patients’ therapeutic responses to the two immune checkpoint inhibitors. The results showed that the IPS values for CTLA-4 positivity and PD-1 positivity in the H-CXCL13 γδT group were significantly higher than those in the low-score group (*p* < 0.001; Figure 7C). The IMvigor210 dataset enrolled urothelial carcinoma patients treated with anti-PD-L1 therapy, which was utilized exclusively for exploratory validation to assess the therapeutic relevance and prognostic potential of our colorectal cancer-derived signature. Such a cross-tumor validation strategy is widely adopted in biomarker research [28,29]. In the IMvigor210 cohort, patients with a high C4_CXCL13 γδT signature score had a significantly longer survival period than those with a low score (*p* = 0.0044; Figure 7D). Meanwhile, patients in the high-score group who underwent anti-PD-L1 treatment achieved a prominently superior objective response rate relative to the low-score group (Chi-square test, *p* = 0.0167; Figure 7E). Correspondingly, the high-score group displayed a higher percentage of complete response and a lower rate of progressive disease (Figure 7F). In conclusion, the results demonstrate that the H-CXCL13 γδT group displays a superior response to immunotherapy relative to the low-score group, and the signature score of C4_CXCL13 γδT cells can be used as a favorable biological factor for predicting the prognosis of immunotherapy.

**Figure 8:**
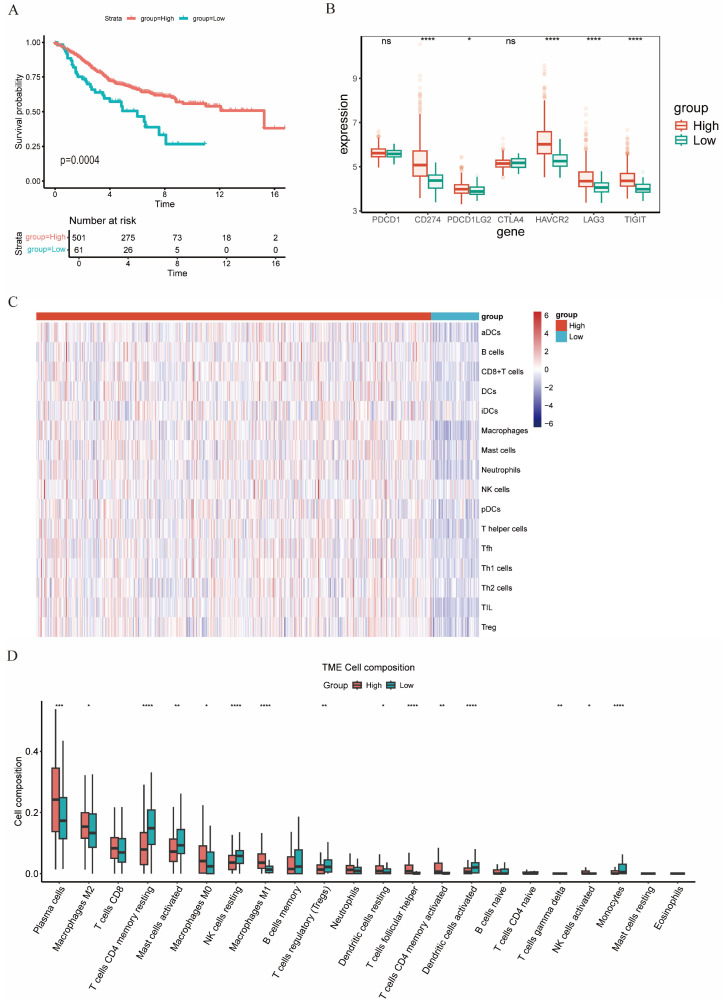
Independent validation of immune signatures across the two groups. (**A**) Prognostic value of C4_CXCL13 γδT cell signature scores in colon cancer. (**B**) Differential expression patterns of immune checkpoints in the two groups. (**C**) Heatmap illustrating the differential immune cell infiltration profiles between H-CXCL13 γδT and L-CXCL13 γδT groups. (**D**) The relative abundance of immune cells between two groups. *p* < 0.05 (*), *p* < 0.01 (**), *p* < 0.001 (***), and *p* < 0.0001 (****).

To externally validate the clinical implications of the C4_CXCL13 signature, 558 colon cancer samples from GSE39582 were scored and grouped similarly to the TCGA cohort. A high C4_CXCL13 γδT score was significantly associated with improved survival relative to a low score (Figure 8A). We subsequently assessed the expression of immune checkpoints and the degree of immune infiltration via ESTIMATE, ssGSEA, and CIBERSORT. With the exception of PD1 and CTLA4, all other immune checkpoints were highly expressed in the H-CXCL13 γδT group (Figure 8B). Immune cell infiltration profiles showed a similar trend to those in TCGA-COAD (Figure 8C,D). Therefore, using GSE39582, we verified that high C4_CXCL13 γδT signature scores are associated with a more active immune microenvironment, thereby predicting a more favorable response to immunotherapy.

## 4. Discussion

γδT cells act as a bridge between innate immunity and adaptive immunity, which are particularly abundant in barrier areas such as the intestines [30]. Emerging evidence has demonstrated that γδT cells serve critical functions in regulating CRC progression; however, their precise roles are debated, largely owing to the heterogeneity of the TME [31]. scRNA-seq analysis allows for the delineation of unique γδT cell subsets in the complex tumor microenvironment, thus providing novel insights for immunotherapeutic interventions. In this study, by integrating single-cell RNA-seq, bulk RNA-seq, and clinical cohorts, we acquired a considerable number of γδT cells and systematically characterized their heterogeneity within the tumor microenvironment of colorectal cancer with distinct microsatellite instability statuses.

Our analysis reveals extensive tumor-associated γδT cells heterogeneity, with distinct subsets exhibiting divergent transcriptional programs and prognostic impacts. Intriguingly, a distinct C4_CXCL13 γδT cell subset was identified, which expresses multiple immune checkpoint molecules such as PD-1 and simultaneously exhibits a cytotoxic T cell phenotype, characterized by elevated expression of genes related to the perforin-granzyme pathway. The C4_CXCL13 γδT subset was significantly enriched in immune response pathways such as “antigen processing and presentation”, “cell killing”, and “PD-1 signaling pathway”. Pseudotime analysis results suggested that the MSI CRC tumor microenvironment drives γδT cells to differentiate from the proliferative C3_TOP2A γδT cells into effector C4_CXCL13 γδT cells and memory C1_IL7R γδT cells, accompanied by enhanced cytotoxic and antigen-presenting functions. T cell exhaustion is a dynamic and progressive process. To date, it remains unclear whether γδT cells exhibit exhaustion in the colorectal cancer tumor microenvironment and what the characteristics of these potential exhausted cells are. However, with in-depth research, it has been found that some T cell subsets can still maintain their effector functions even when displaying an exhausted phenotype. It has been previously documented that the Vδ2^−^ subset of γδT cells in human renal carcinoma, although phenotypically similar to exhausted T cells, still retains cytotoxic functions. Its expression characteristics are correlated with the clinical response to PD-1 inhibitors [32] and could be utilized as a predictive biomarker for immune checkpoint blockade. Based on this, we speculate that the C4_CXCL13 γδT subset with similar functional characteristics may be one of the important cellular bases for MSI CRC patients to benefit from immune checkpoint blockade therapy. In addition, PD-1-expressing tumor-infiltrating γδT cells have also been found in neuroblastoma [33] and pancreatic ductal adenocarcinoma [34]. Furthermore, we found that C4_CXCL13 γδT cells are almost absent in normal colorectal tissues and their proportion is higher in MSI patients than in MSS patients. Therefore, we explored their role in tumor progression and immunotherapy of MSI CRC.

Further analysis of C4_CXCL13 γδT cell-related cell communication revealed that this cell subset interacts between this subset and tumor cells via the IFNG-(IFNGR1 + IFNGR2), GZMA-F2RL1, and GZMA-PARD3 signaling pathways, indicating that C4_CXCL13 γδT cells possess cytotoxic activity. Among these predicted interactions, the GZMA-F2RL1 signaling pair was identified as a prominent candidate pathway between C4_CXCL13 γδT cells and tumor cells, and F2RL1 is specifically highly expressed only in CRC tumor cells, serving as an important molecule for the interaction between tumor cells and C4_CXCL13 γδT cells. The *F2RL1* gene encodes the protease-activated receptor 2 (PAR2) protein, which is classified as a G protein-coupled receptor. PAR2 has been reported to be abnormally activated in a wide range of malignant tumor types, such as colon cancer [35] and breast cancer [36] and plays a critical role in regulating tumor cell migration, drug resistance, angiogenesis, and other malignant biological behaviors. PAR2 is highly expressed in colorectal cancer cells and fosters an immunosuppressive tumor microenvironment by suppressing type I interferon signaling, whereas its inhibition enhances anti-tumor immunity, increases CD8^+^ T cell infiltration in metastatic lesions, and synergizes with immune checkpoint blockade to suppress liver metastasis [37]. While these interactions are computationally inferred, given the known oncogenic roles of F2RL1 (PAR2) in the tumor microenvironment, we speculate that immunotherapy targeting the GZMA-F2RL1 signaling pathway holds great promise.

We identified markers of C4_CXCL13 γδT cells and ultimately determined 17 relevant genes exhibiting associations with MSI and immune processes (*TRGC2*, *S100A4*, *CD3D*, *GZMB*, *CD3G*, *LAG3*, *HLA-DPB1*, *CD2*, *HLA-DRA*, *CCL4*, *RBPJ*, *GNLY*, *HLA-DPA1*, *CYTOR*, *IDH2*, *HLA-DRB1*, *PTMS*). We constructed a C4_CXCL13 γδT cell signature score via GSVA, and stratified samples into low-CXCL13 and high-CXCL13 γδT groups. Patients with a high C4_CXCL13 γδT score exhibited better prognosis, and its prognostic value was further validated in two additional independent datasets (IMvigor210, GSE39582). We then evaluated clinical characteristics and demonstrated that the C4_CXCL13 γδT score serves as an independent prognostic marker. Gene set enrichment analysis revealed that genes elevated in the high C4_CXCL13 γδT signature group were markedly enriched in pathways associated with MHC molecules. Moreover, cell–cell communication analysis revealed the activation of ligand–receptor pairs involved in antigen presentation and immune cell recruitment during interactions between γδT cells and other immune populations. Accumulating evidence has indicated that γδT cells can serve as antigen-presenting cells (APCs). Activation of these cells leads to increased expression of MHC-II and costimulatory molecules, ultimately promoting the priming of naive αβT cells and B cells [38,39]. Notably, γδT cells can promote macrophage activation, and their coordinated function may contribute to synergistic antitumor immune effects [40]. Consistent with the above results, our analysis demonstrates that γδT cells could enhance the accumulation of macrophages within the tumor microenvironment via chemokine-dependent signaling.

To further characterize the distinct immune functional profiles between H-CXCL13 γδT and L-CXCL13 γδT groups, we employed ESTIMATE, ssGSEA, and CIBERSORT. ESTIMATE analysis indicated that stromal scores, immune scores, and ESTIMATE scores were significantly elevated in the H-CXCL13 γδT group. ESTIMATE analysis revealed that the H-CXCL13 γδT group exhibits significantly higher stromal scores, immune scores, and ESTIMATE scores. Subsequent CIBERSORT analysis indicates that the percentages of CD8^+^ T cells and M1 macrophages were significantly elevated in the H-CXCL13 γδT group compared with the L-CXCL13 γδT group. CD8^+^ tumor-infiltrating lymphocytes (TILs) contribute to tumor suppression through the recognition of tumor-specific antigens [41]. M1 macrophages also exert antitumor roles through direct cytotoxicity and antibody-dependent cell-mediated cytotoxicity (ADCC) to eradicate tumor cells. These results collectively imply that increased immune infiltration, especially CD8^+^ T cells and M1 macrophages, is observed in the H-CXCL13 γδT group, which may represent a beneficial predictor for immunotherapeutic response in CRC. This observation may be attributed to the heterogeneity of tumor immunophenotypes. In light of the diverse immune infiltration profiles, human cancers can be stratified into inflamed, immune-excluded, and immune-desert subtypes [42]. Inflamed tumors generally possess a higher level of immune cell infiltration, whereas immune-desert tumors display low immune infiltration. Moreover, immune cell infiltration levels are positively correlated in bulk-sequencing data. Therefore, C4-CXCL13 γδT cells may infiltrate the tumor microenvironment together with CD8^+^ T cells and M1 macrophages, leading to a correlation between high H-CXCL13 γδT scores and increased immune infiltration as well as better immunotherapy responsiveness.

To investigate the association of C4-CXCL13 γδT scores with immunotherapeutic response, we analyzed the mutation profiles of the two groups. Tumor mutational burden (TMB) was calculated for each sample, revealing markedly higher TMB levels in the H-CXCL13 γδT group compared with the low group. Neoantigens arise from tumor mutations, and higher TMB is thus accompanied by increased neoantigen load. This not only facilitates T cell recognition but also corresponds to superior clinical benefits from immunotherapy [43]. We next performed a comparative analysis of immune checkpoint expression between the two groups [44]. High expression of these immune checkpoints provides a suitable basis for the utilization of ICIs, thereby contributing to improved clinical efficacy in immunotherapy [45]. The predictive potential of the C4_CXCL13 γδT score was further assessed in an immunotherapy cohort. A high H-CXCL13 γδT score was found to be associated with improved therapeutic efficacy of both CTLA-4 and PD-1 immune checkpoint inhibitors. The analysis of the IMvigor210 dataset revealed a higher complete response (CR) rate and a lower progressive disease (PD) rate in the H-CXCL13 γδT group than in the L-CXCL13 γδT group. These findings collectively demonstrate that the H-CXCL13 γδT group displays a more favorable response to immunotherapy. To validate the association between C4_CXCL13 γδT scores and immunotherapeutic response, samples in GSE39582 were classified into two groups according to C4_CXCL13 γδT scores. We further evaluated immune checkpoint transcript levels and immune cell infiltration patterns. Our analysis demonstrated that the majority of immune checkpoints were highly expressed in the H-CXCL13 γδT group. Furthermore, the patterns of immune cell infiltration were in accordance with those observed in the TCGA-COAD cohort. Thus, analysis of the GSE39582 cohort confirmed that the H-CXCL13 γδT group may exhibit superior immunotherapeutic responsiveness compared with the L-CXCL13 γδT group.

This study has several limitations. First, although cohort-specific optimal cutoff values identified using the “surv_cutpoint” function have been widely adopted in previous studies to account for data heterogeneity, this approach may introduce overfitting and optimistic bias, potentially leading to an overestimation of prognostic performance. Thus, the present results should be interpreted with appropriate caution. Second, the IMvigor210 cohort was employed solely for exploratory validation of our colorectal cancer-derived signature. Given the inherent tissue-of-origin difference, future validation in independent CRC immunotherapy cohorts is warranted.

## 5. Conclusions

In conclusion, our study identified C4_CXCL13 γδT cells that were enriched in CRC patients with microsatellite instability. We further screened 17 genes linked to C4_CXCL13 γδT cells and established C4_CXCL13 γδT cell scores that enable effective prediction of immunotherapy responsiveness in COAD patients. Compared with the L-CXCL13 γδT group, the H-CXCL13 γδT group displays enhanced immune infiltration, elevated TMB, and higher expression of immune checkpoints, and achieves superior immunotherapeutic outcomes. Collectively, these observations advance our understanding of tumor immunity and represent a potential clinical tool for optimizing immunotherapy regimens in COAD.

## Figures and Tables

**Table 1:** Prognostic value of clinical variables evaluated by univariate and multivariate Cox analyses in TCGA-COAD.

Variables	Univariate Analysis		Multivariate Analysis	
	HR (95% CI)	*p* Value	HR (95% CI)	*p* Value
Gender	0.94 (0.56–1.56)	0.808	0.73 (0.43–1.22)	0.229
Age	1.7 (0.9–3.2)	0.102	2.38 (1.23–4.61)	0.010
T	6.6 (1.61–27.1)	0.00887	3.72 (0.88–15.70)	0.074
M	4.01 (2.29–7.03)	1.24e–06	2.71 (1.47–5.00)	0.001
N	3.5 (2.06–5.97)	3.94e–06	2.73 (1.51–4.91)	0.001
C4_CXCL13 γδT score	0.50 (0.30–0.85)	0.0095	0.52 (0.31–0.90)	0.018

## Data Availability

Publicly available datasets were analyzed in this study. This data can be found here: http://www.ncbi.nlm.nih.gov/geo/ (accessed on 20 January 2026), accession number: GSE178341, GSE39582; http://www.cancer.gov/tcga (accessed on 20 January 2026).

## Supplementary Materials

The following supporting information can be downloaded at: https://www.mdpi.com/article/10.3390/genes17040387/s1. Figure S1: Quality control of single-cell RNA-seq data in colorectal cancer. Figure S2: Mutation profile landscapes of the L-CXCL13 γδT and H-CXCL13 γδT groups. Figure S3: Immune infiltration and immune checkpoint expression profiles between H-CXCL13 γδT and L-CXCL13 γδT groups in MSI and MSS CRC patients. Table S1: The microsatellite statuses of all the patients in GSE178341 dataset. Table S2: Differentially expressed genes in each γδT cell clusters.
