# Supplementary material for: Single-Cell Sequencing Reveals γδT Cell Heterogeneity Under Distinct Microsatellite Statuses as a Potential Biomarker for Immunotherapy and Prognosis in Colorectal Cancer
Source: Genes (Basel). 2026 Mar 29;17(4):387. doi: 10.3390/genes17040387 (PMC13116210; doi:10.3390/genes17040387)
Supplement: Supplementary file 1 [file genes-17-00387-s001.zip › Supplementay Figure.pdf]

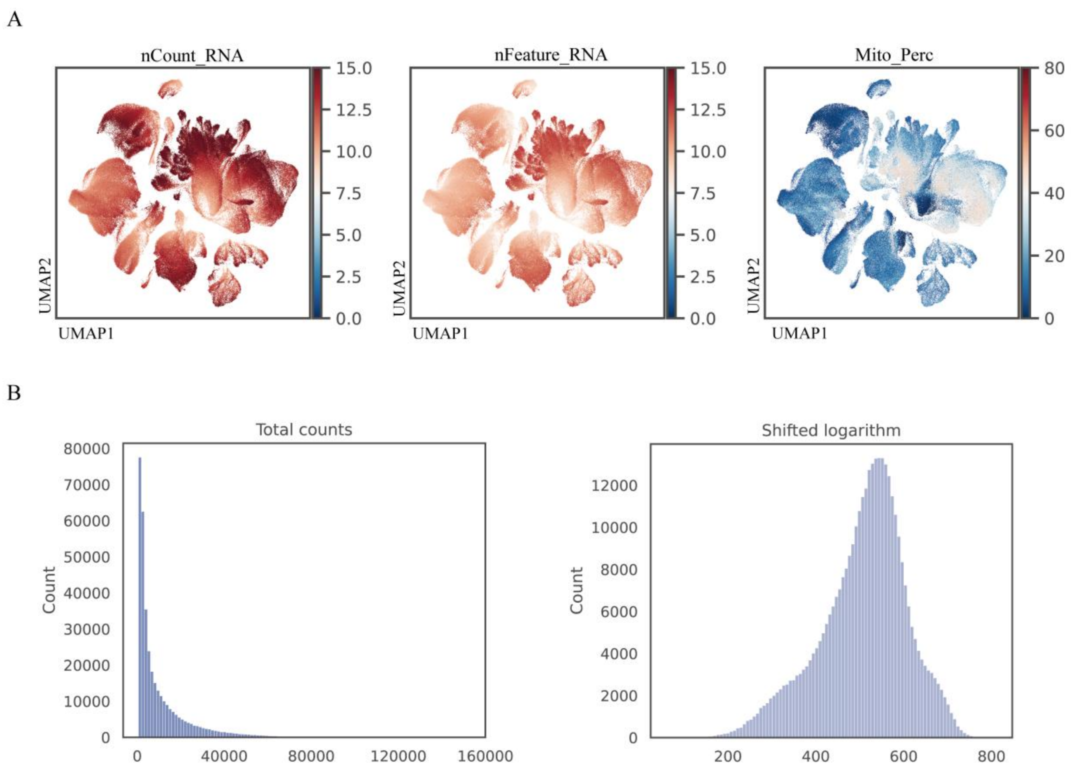

Figure S1. (A) UMAP plot of the number of counts (nCount_RNA), number of gene (nFeature_RNA), and percentage of mitochondrial genes (Mito_Perc) for quality control of the GSE178341 dataset. (B) Distribution of gene expression levels per cell before and after normalization.

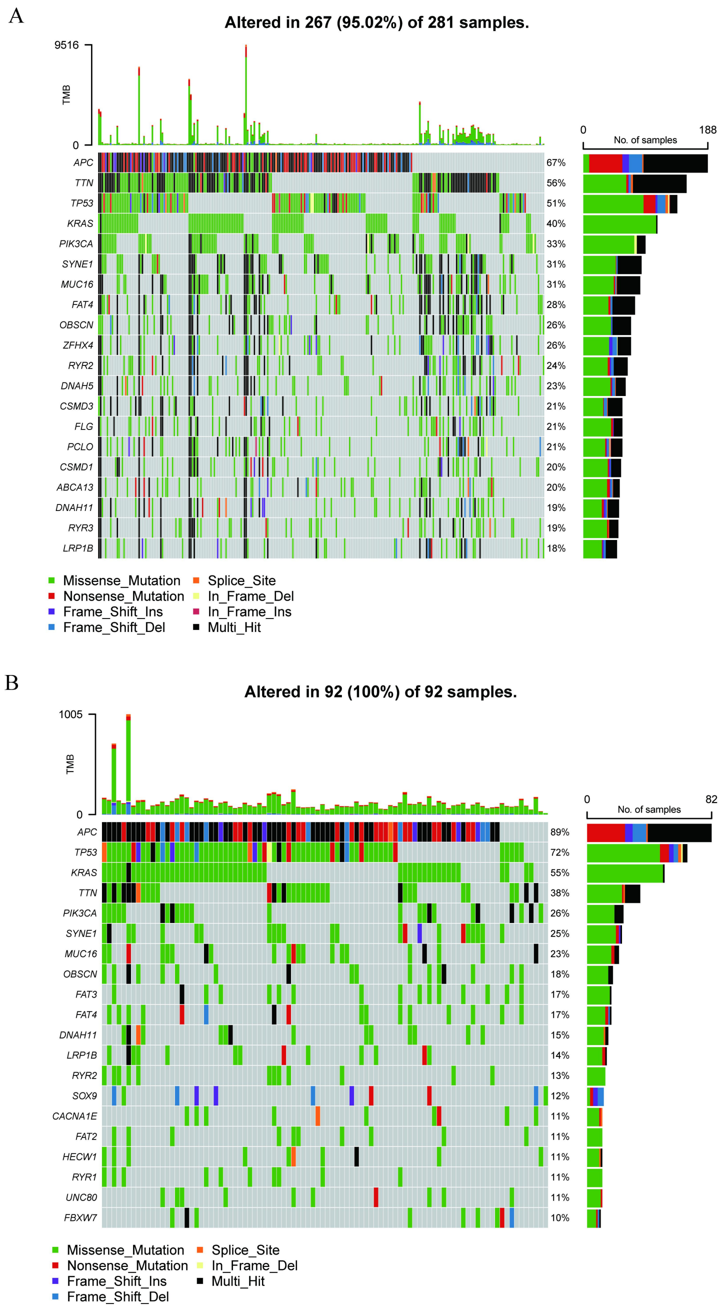

Figure S2. (A) Landscapes of mutation profiles of H-CXCL13 γδT group. (B) Landscapes of mutation profiles of L-CXCL13 γδT group.

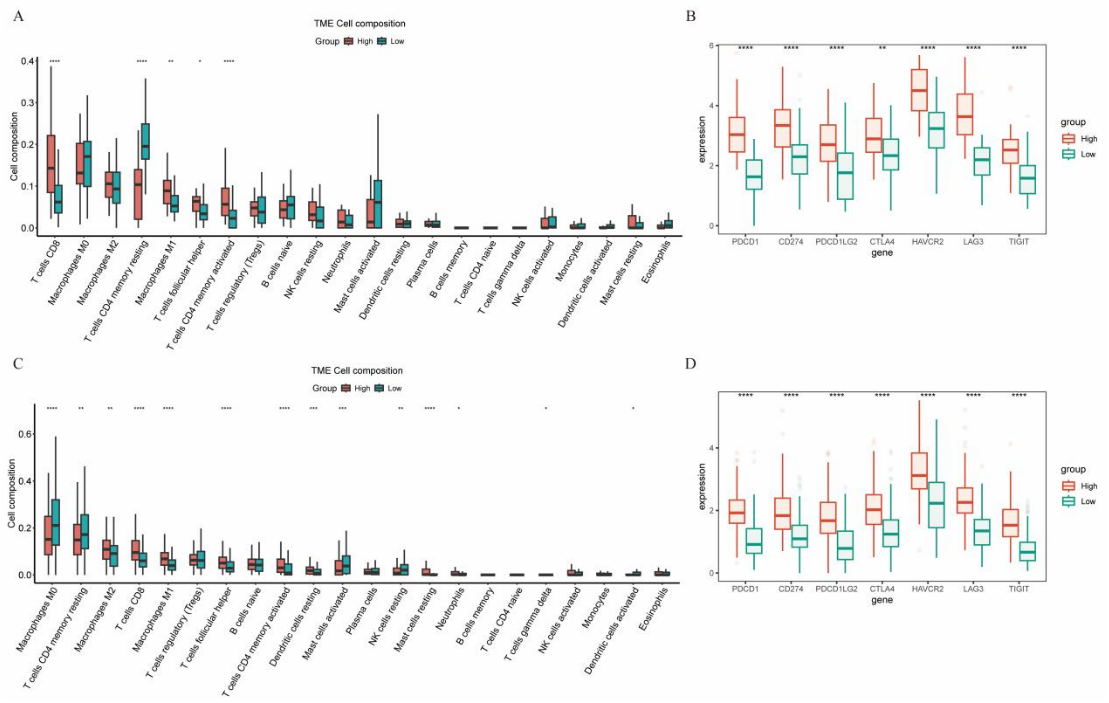

Figure S3. (A) Differences in immune cell infiltration between the high and low score groups in patients with MSI. (B) Expression differences in the immune-checkpoint genes between the high and low groups in patients with MSI. (C) Differences in immune cell infiltration between the high and low score groups in patients with MSS. (D) Expression differences in the immune-checkpoint genes between the high and low groups in patients with MSS.
